# Maternal Prenatal Supplements Intake and Dietary Sources and Their Relation to Autism Spectrum Disorder in the Population of Bangladesh

**DOI:** 10.1002/puh2.70253

**Published:** 2026-04-27

**Authors:** Md. Shahid Khan, Mohammad Alamgir Kabir, Shafi Mohammad Tareq

**Affiliations:** ^1^ National Institute of Traumatology and Orthopedic Rehabilitation Dhaka Bangladesh; ^2^ Center for Physiotherapy and Rehabilitation Chattogram Bangladesh; ^3^ Department of Environmental Sciences Jahangirnagar University Savar, Dhaka Bangladesh; ^4^ Department of Statistics and Data Science Jahangirnagar University Savar, Dhaka Bangladesh

**Keywords:** autism spectrum disorder, dietary source, food safety, health risk, prenatal supplement

## Abstract

Studies on maternal prenatal supplements intake and sources of dietary factors related to the risk of autism spectrum disorder (ASD) are in their infancy, especially in developing countries like Bangladesh. An observational case–control study in Bangladesh delved into the potential connections between prenatal nutrition and the likelihood of ASD. Parents of 310 ASD cases and 310 healthy controls (HCs) participated, sharing insights through structured questionnaires and face‐to‐face interviews. This study focused on associations with significant levels at *p* ≤ 0.05, employing odds ratios (ORs) and 95% confidence intervals (CIs) to pinpoint potential risk factors. The findings offer intriguing insights as follows: consuming prenatal supplements, farmed chicken or eggs, and farmed vegetables during pregnancy and the first 3 years of life emerged as associated with a reduced likelihood of ASD compared to HCs. Interestingly, the opposite trend was observed for farmed fish and fruits during the same period, alongside maternal substance or drug misuse during pregnancy, which was linked to increased ASD odds. Additionally, higher parental socioeconomic status during pregnancy appeared to have a protective effect, whereas the male gender emerged as a significant risk factor. Considering the substantial public health and economic burden of ASD, these findings highlight potential avenues for intervention. Encouraging responsible prenatal supplement intake, discouraging maternal substance or drug misuse, and ensuring the safety of farmed fish, chicken or eggs, fruits and vegetables during pregnancy and early childhood may offer valuable tools for potentially reducing ASD risk.

## Graphical abstract

Consuming prenatal supplements, farmed chicken/eggs, and vegetables during pregnancy and the first 3 years of life showed protective relation with ASD compared with healthy controls. Consuming farmed fish and fruits during the same period, alongside maternal substance/drug misuse during pregnancy linked to increased ASD odds.

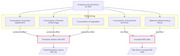

## 1 Introduction

Autism spectrum disorder (ASD) is a complex neurodevelopmental disability in early childhood [1], characterized by persistent deficits in social communication and social interaction across multiple contexts, accompanied by restricted, repetitive patterns of behavior, interests, or activities [2]. Although genetic predisposition plays a pivotal role, research increasingly highlights the modifying influence of various prenatal and postnatal environmental factors (EFs) on ASD‐linked genes [3, 4]. These nongenetic contributors, encompassing reproductive, dietary, chemical, and demographic exposures, are collectively termed environmental risk factors for ASD [5]. Understanding ASD's etiology likely demands acknowledging the intricate interplay between genetic, epigenetic, neurological, hormonal, and environmental influences, which often synergistically contribute to its emergence [6].

The intricate link between maternal diet and offspring neurodevelopment is undeniable [7]. Given associations between ASD and maternal metabolic dysfunction, and the impact of dietary habits on maternal health and risk of metabolic diseases [8], prenatal dietary patterns emerge as a crucial area of investigation in ASD etiology. The risk of ASD was linked to maternal dietary factors, where only 81% of mothers having an ASD child received an adequate diet during pregnancy as compared with 93% of controls [9]. Indeed, diet, a key component in brain development and neuropsychiatric diseases, can have an impact on fetal serotonin (5‐HT) levels throughout gestation [10]. A meta‐analysis of a prospective cohort found that maternal folic acid or multivitamin supplementation is associated with a decrease in the risk of children's ASD. However, relationships between other dietary‐related factors and ASD were indecisive [11]. Furthermore, there have been few studies conducted about ASD in Bangladesh [12, 13, 14]. Therefore, more research is necessary to completely understand the importance of maternal dietary patterns in ASD [11, 15]. Food adulteration (mixing, deception, and substitution of various hazardous substances for high‐quality ingredients in the production of food) is detrimental for public health in Bangladesh [16]. This study aimed to assess the relationships between the likelihood of ASD and maternal prenatal supplement intake and the sources of some dietary factors in the Bangladeshi population.

## 2 Methods

### 2.1 Study Design

This study was an observational case–control survey.

### 2.2 Participants (Inclusion and Exclusion Criteria)

This study utilized a case–control design, incorporating participants from across all Bangladeshi divisions. Cases were registered individuals with ASD identified by Protibandhi Sheba O Shahajjo Kendro (PSOSK), a disability support and service center under the Ministry of Social Welfare. The diagnosis of ASD was conducted prior to this study by the relevant specialist team of PSOSK using the “Diagnostic and Statistical Manual of Mental Disorders, Fifth Edition (DSM‐5).” The DSM‐5 was used in a consistent manner for all the ASD cases. Healthy controls (HCs) were recruited from relatives (except same immediate family) and friends residing in the same divisions as the ASD cases, following established methods employed by [17, 18]. Focusing on parents as respondents ensured access to the most comprehensive information about the subjects, surpassing knowledge levels typically held by relatives, caregivers, or foster/stepparents [19]. Unwilling to consent (36 parents of ASD individuals who declined consent), foster/stepparents (two), and those with a diagnosed psychiatric illness (two) or deafness (three) were excluded for case group. Income disparities emerged between groups, with the monthly total household income (MTHI) during pregnancy ranging from 1075 to 600,000 Bangladeshi taka (BDT). Median incomes were 20,000 and 15,000 BDT for the control and case groups, respectively. Parental age data also revealed differences, with fathers’ and mothers’ ages at the subject's birth spanning 15–63 and 12–48 years, respectively, whereas subjects themselves ranged from 2 to 24 years old.

### 2.3 Materials

The structured questionnaire was constructed after an extensive review of the literature [5, 6, 9, 11, 12, 13, 14, 20, 21, 22, 23, 24, 25, 26] and Bangladeshi perspectives with a focus on prenatal supplements and sources of some dietary factors (Table 4), including a few sociodemographic factors (SDFs) (Tables 2 and 3) that might be related to the likelihood of ASD in Bangladesh. The questionnaire was validated by experts from academics (supervisors and biostatistician), practitioners (pediatricians, physicians, and disability specialists), and a researcher specializing in autism, guided by the literature [27]. The questionnaire was pretested in Dhaka and Chattogram divisions on 30 parents having a child with ASD.

### 2.4 Measures

Prenatal supplementary drug intake and misuse of substances or drugs during pregnancy are categorized as no (reference group = RG) and yes. During pregnancy to postnatal 3 years: (1) The dichotomous food source variables (consumed chicken or egg, vegetables, fruits, and fish) are categorized as farmed and others. Others were RG, which generally refers to foods sourced from natural, wild, or noncommercial environments. These represent foods directly obtained from natural, untamed ecosystems, often referred to as wild‐caught, gathered, or hunted items. This includes wild fish/seafood, foraging, hunting for meat, and uncultivated fruits or vegetables. In addition, farmed items were raised or cultivated in controlled environments, including livestock farms, poultry houses, fish farms (aquaculture), and commercial crop farming (vegetables/fruits). (2) Additionally, within the same periods, drinking arsenic‐ or iron‐free water, filtering water before drinking, consuming soft drinks, ingesting processed juice, and consuming frozen food (refers to items that have been preserved in refrigerator) are categorized as “no” (RG) or “yes.” Correspondingly, some sociodemographic covariates (SDFs) were also assessed across various categories: (1) MTHI during pregnancy was used to measure socioeconomic status (SES) and categorized as follows: ≤10,000 (RG), 10,001–20,000, and ≥20,001 BDT. (2) Level of education during pregnancy for both father and mother, segmented into three categories: ≤Primary (RG), SSC/HSC, and ≥bachelor's degree. (3) Age during childbirth is categorized into three groups {≤21 years (RG), 22–35 years, and ≥36 years for fathers; and ≤18 years (RG), 19–30 years, and ≥31 years for mothers}. (4) The subject's gender was categorized as female (RG) and male. The subject's age was not a variable of interest in this case–control study, as it is not the primary exposure being studied. Because the researchers recruited the sample of this study from the register book of PSOSK, where the individual with autism was diagnosed previously by the PSOSK clinical team. The age of parents during child birth is very important and was discussed well.

### 2.5 Procedures

A total of 24 out of 103 PSOSK centers were included by a simple random sampling method throughout Bangladesh, covering each division, to collect case data (Table 1). By using a table of sample size [28], 306 individuals with ASD were selected as cases, and a similar number of HCs were selected from the same areas and ages of the cases [17, 18]. Finally, 310 cases from the 1498 population of ASD and a similar number of HCs from relatives and friends were recruited randomly. During data collection, held from January 2020 to June 2021, rigorous scientific procedures were adhered to. Trained personnel employed standardized protocols and provided clear explanations to all respondents. Written informed consent was obtained before proceeding with face‐to‐face interviews, and strict confidentiality of data was maintained throughout the process.

**TABLE 1:** Sampling method for population of autism spectrum disorder (ASD).

Simple random sampling formula to select the PSOSK^a^	Division wise total PSOSK	Selected PSOSK by simple random sampling	Registered number of ASD at selected PSOSK	Approximate % to be included from total population of ASD
Five: ≥20 PSOSK	Dhaka, 24	5	308	20
Four: 15–19 PSOSK	Chattogram, 18	4	304	20
Three: 10–14 PSOSK	Rajshah, 10	3	332	20
	Rangpur, 14	3	136	10
	Khulna, 13	3	145	10
Two: ≤9 PSOSK	Mymensingh, 7	2	84	5
	Barisal, 9	2	55	5
	Sylhet, 8	2	134	10
**Total**	103	24	1498	100

^a^ PSOSK, Protibandhi Sheba O Shahajjo Kendro, a disability support and service center under the Ministry of Social Welfare.

### 2.6 Statistical Analyses

**FIGURE 1:**
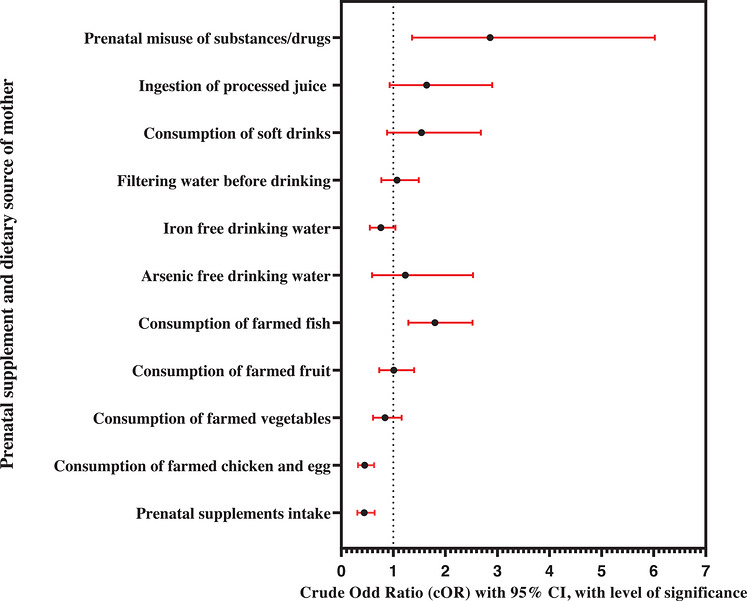
Forest plot showing the unadjusted or crude odds ratios (cORs) with 95% confidence intervals (CIs) and level of significance (*p* value) for the relationships between autism and prenatal supplement and dietary source of mother during pregnancy to postnatal 3 years in the cases and controls, calculated by binary logistic regression (BLR) model analysis, recruited from Bangladesh, 2020–2021 (*n* = 620).

**FIGURE 2:**
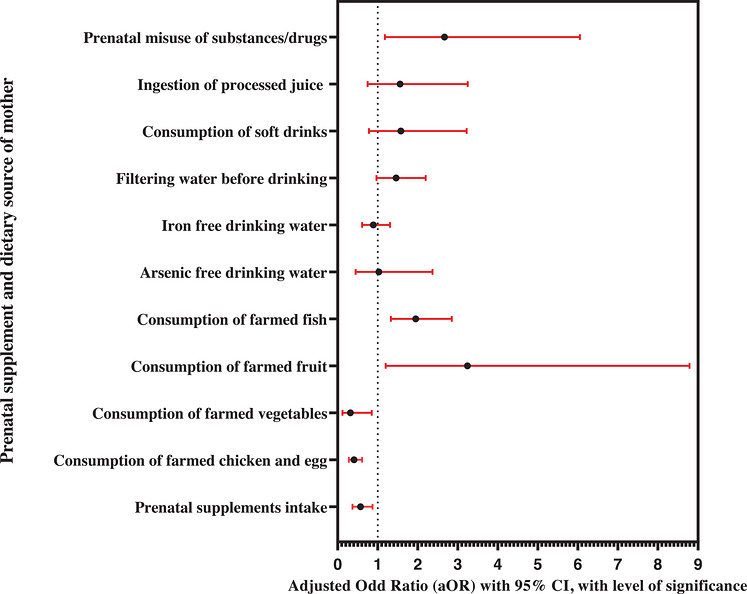
Forest plot showing the adjusted odds ratios (aORs) with 95% confidence intervals (CIs) and level of significance (*p* value) for the relationships between autism and prenatal supplement and dietary source of mother during pregnancy to postnatal 3 years in the cases and controls, calculated by multiple logistic regression (MLR) model analysis, recruited from Bangladesh, 2020–2021 (*n* = 620). MLR model adjusted for paternal and maternal ages at child's birth, and their levels of education and SES during pregnancy, and subject's gender.

IBM SPSS version 23 was utilized for data analysis. Univariate analysis employed appropriate measures like number (percentage), mean ± standard deviation (SD), or median to describe the data. In bivariate analysis, the independent sample *t*‐test and nonparametric tests were utilized to compare mean and median values, respectively, whereas the chi‐square test and cross‐tabulation were employed for independent variables (IVs) with the participant group (dependent variable, DV). Statistical significance was set at *p* ≤ 0.05. Odds ratios (ORs) within 95% confidence intervals (CIs) were calculated to identify potential risk factors. The binary LR (BLR) analysis {for crude/unadjusted OR (cOR)} was done to observe the association between the development of ASD among subjects and each of the covariates individually (Table 4 and Figure 1). On the basis of the BLR analysis and a priori information, a multiple LR (MLR) model was constructed to examine the relationship between variables (significant in BLR) and the odds of ASD development for the adjusted OR (aOR) and model fitness. The MLR model adequately fitted the data after adjusting the covariates. The MLR model was developed for prenatal supplements and maternal dietary sources, excluding the consumption of frozen food due to its very close frequency between case and control (i.e., the consumption of frozen foods was 14.84% for case group and 15.16% for control group). To adjust the covariate effects, paternal and maternal age groups at the child's birth, their levels of education and SES during pregnancy, and the subject's gender were added using the enter method. The full MLR model (Table 5 and Figure 2) in Omnibus Tests containing all predictors was statistically significant, *X* ^2^ (22, *N *= 620) = 126.02, *p < *0.001, indicating that the full model was able to distinguish between case and control. On the basis of the statistical test for goodness of fit, the full MLR model analysis fit the data adequately by the Hosmer and Lemeshow test, and it was better than the 0 block model, and it was statistically predicted correctly: *X* ^2^ (8, *N *= 620) = 8.05, *p *= 0.429. The full model as a whole explains between 18.4% (Cox and Snell *R* Square) and 24.5% (Negelkerke *R* Square) of the variance in subject status and correctly classified 70.2% of subjects. The subject's age was excluded from the LR analysis as it was not a variable of interest.

### 2.7 Patient and Public Involvement

Direct involvement of patients in formulating research questions, selecting outcome measures, or contributing to the design and implementation of the study did not occur. They were also not asked for advice on the interpretation or writing of the results. However, parents voluntarily participated in the interviews and received periodic updates on the study's progress and outcomes.

### 2.8 Research Ethics and Patient Consent

This study was in accordance with the Declaration of Helsinki [29] and approved by the “Higher Studies Committee” (Serial number: 45; Reg. Number: 2931; date: March 6, 2018), and “Biosafety, Biosecurity and Ethical Committee” {Ref No: BBEC.JU/M 2023/01(12)} of the Jahangirnagar University. Stringent measures were implemented to ensure confidentiality of both individuals and information. Written informed consent was taken from respondents before data collection.

## 3 Results

### 3.1 Sociodemographic Factors (SDFs) or Covariate Characteristics

The SDF's statistics and characteristics obtained by univariate and bivariate analyses are presented in Tables 2 and 3. The MTHI during pregnancy ranged from 1075 to 600,000 taka with a mean of 27,921.4919 and an SD of ±40,179.43351. The father's and mother's ages at the subject's birth ranged from 15 to 63 years with a mean of 32.7581 and an SD of ±6.96607; 12–48 years with a mean of 24.4290 and a SD of ±5.84643, respectively; and 2–24 years with a mean of 10.0468 and an SD of ±5.40532 for subjects. The chi‐square [*X* ^2^] test and cross‐tabulation revealed a significant association with ASD for all of the six SDFs except the mother's age group (*p* = 0.114) (Table 3).

**TABLE 2:** The distribution of age, monthly total household income (MTHI), and division in the autism spectrum disorder (ASD) cases and healthy controls, recruited from Bangladesh, 2020–2021 (*n* = 620).

Factors	Healthy control (310)	ASD (310)	All (620)	*p* value
Subjects’ age in year (mean ± SD)	9.8 ± 6.06	10.3 ± 4.65	10.05 ± 5.41	0.25
Fathers’ age^a^ (mean ± SD)	32.83 ± 6.66	32.69 ± 7.27	32.76 ± 6.97	0.81
Mothers’ age^a^ (mean ± SD)	24.97 ± 5.79	23.88 ± 5.86	24.43 ± 5.85	**0.02**
MTHI^b^ during pregnancy (median) in BDT^c^	20,000	15,000	18,000	**<0.001**
Division of the respondent, *n* (%)				
Chattagram	57 (18.4)	74 (23.9)	131 (21.1)	**0.002**
Barisal	21 (6.8)	21 (6.8)	42 (6.8)	
Rajshahi	30 (9.7)	52 (16.8)	82 (13.2)	
Khulna	23 (7.4)	20 (6.5)	43 (6.9)	
Mymensingh	33 (10.6)	9 (2.9)	42 (6.8)	
Sylhet	29 (9.4)	26 (8.4)	55 (8.9)	
Rangpur	59 (19.0)	49 (15.8)	108 (17.4)	
Dhaka	58 (18.7)	59 (19.0)	117 (18.9)	

*Note:* Bold *p* values represent the significant relation.

^a^ At the time of child's birth, in year.

^b^ MTHI = Monthly total household income, determinant for socioeconomic status (SES).

^c^ BDT = Bangladeshi taka.

**TABLE 3:** The frequency distribution and binary logistic regression (BLR) analysis between autism spectrum disorder (ASD) risk and sociodemographic factors (SDFs) in the cases and controls, recruited from Bangladesh, 2020–2021 (*n* = 620).

	Cross tabulation (*n*, %) with *X^2^*‐test			BLR	
Socio‐demographic factors	Healthy control (310)	ASD (310)	*p*‐value	cOR (95%CI)	*p*‐value
Father's education^a^			**0.002**		
≤Primary	113 (36.5)	130 (41.9)		Reference	
SSC/HSC	47 (15.2)	71 (22.9)		1.31 (0.84, 2.05)	0.232
≥Bachelor degree	150 (48.4)	109 (35.2)		0.63 (0.44, 0.90)	**0.011**
Mother's education^a^			**0.001**		
≤Primary	122 (39.4)	143 (46.1)		Reference	
SSC/HSC	78 (25.2)	98 (31.6)		1.07 (0.73, 1.57)	0.722
≥Bachelor degree	110 (35.5)	69 (22.3)		0.54 (0.36, 0.79)	**0.001**
MTHI^a^			**<0.001**		
≤10,000 BDT	71 (22.9)	128 (41.3)		Reference	
10,001‐20,000 BDT	96 (31.0)	83 (26.8)		0.48 (0.32, 0.73)	**<0.001**
≥20,001 BDT	143 (46.1)	99 (31.9)		0.38 (0.26, 0.57)	**<0.001**
Age group of father in year^b^			**0.029**		
≤21	3 (1.0)	10 (3.2)		Reference	
22‐35	228 (73.5)	203 (65.5)		0.27 (0.70, 0.98)	**0.047**
≥36	79 (25.5)	97 (31.3)		0.37 (0.10, 1.38)	0.139
Age group of mother in year^b^			0.114		
≤18	42 (13.5)	61 (19.7)		Reference	
19‐30	224 (72.3)	205 (66.1)		0.63 (0.41, 0.98)	**0.038**
≥31	44 (14.2)	44 (14.2)		0.69 (0.39, 1.22)	0.202
Subject's gender			**<0.001**		**<0.001**
Female	147 (47.4)	91 (29.4)		Reference	
Male	163 (52.6)	219 (70.6)		2.17 (1.56, 3.02)	

*Note:* Bold *p* values represent the significant relation.

Abbreviations: cOR, crude odds ratio; MTHI, monthly total household income.

^a^ During pregnancy.

^b^ At the time of child's birth.

In BLR of SDFs (Table 3), ≥bachelor's degree for level of father's and mother's education as compared to ≤primary, father's age group of 22–35 years as compared to ≤21 years; mother's age group of 19–30 as compared to ≤18 years, and MTHI of 10,001–20,000 and ≥20,001 as compared to ≤10,000 BDT were associated with reduced in the odds of ASD. Male gender, however, was associated with a two‐fold increased odds of ASD in offspring (cOR = 2.17, 95% CI 1.56, 3.02, *p *< 0.001) compared to female.

### 3.2 Prenatal Supplements and Sources of Dietary Factors

Table 4 represents the chi‐square test on prenatal supplements and sources of some dietary factors in the ASD cases and HCs. The prenatal supplementary drug intake, the consumption of farmed chicken, eggs, and fish during pregnancy and the postnatal 3 years, and maternal misuse of substances or drugs during pregnancy were all significantly (*p *≤ 0.05) associated with ASD.

**TABLE 4:** The frequency distribution (cross tabulation with chi‐square test) and binary logistic regression (BLR) analysis between autism and prenatal supplements and sources of some dietary factors during pregnancy to postnatal 3 years in the cases and controls, recruited from Bangladesh, 2020–2021 (*n* = 620).

	Cross tabulation (*n*, %) with *X^2^*‐test			BLR model	
Factors	Healthy control (310)	ASD (310)	*p*‐value	cOR (95%CI)	*p*‐value
Prenatal supplements intake			**<0.001**		
0. No	60 (19.4)	109 (35.2)		Reference	
1. Yes	250 (80.6)	201 (64.8)		0.44 (0.31, 0.64)	**<0.001**
Source of consumed chicken and egg^a^			**<0.001**		
0. Others	81 (26.1)	137 (44.2)		Reference	
1. Firmed	229 (73.9)	173 (55.8)		0.45 (0.32, 0.63)	**<0.001**
Source of consumed vegetable^a^			0.286		
0. Others	116 (37.4)	129 (41.6)		Reference	
1. Firmed	194 (62.6)	181 (58.4)		0.84 (0.61, 1.16)	0.286
Sources of consumed fruit^a^			0.934		
0. Others	117 (37.7)	116 (37.4)		Reference	
1. Firmed	193 (62.3)	194 (62.6)		1.01 (0.73, 1.40)	0.934
Sources of consumed fish^a^			**0.001**		
0. Others	223 (71.9)	182 (58.7)		Reference	
1. Firmed	87 (28.1)	128 (41.3)		1.80 (1.29, 2.52)	**0.001**
Arsenic free drinking water^a^			0.580		
0. No	17 (5.5)	14 (4.5)		Reference	
1. Yes	293 (94.5)	296 (95.5)		1.23 (0.59, 2.53)	0.581
Iron free drinking water^a^			0.086		
0. No	115 (37.1)	136 (43.9)		Reference	
1. Yes	195 (62.9)	174 (56.1)		0.76 (0.55, 1.04)	0.086
Filtering water before drinking^a^			0.676		
0. No	200 (64.5)	195 (62.9)		Reference	
1. Yes	110 (35.5)	115 (37.1)		1.07 (0.77, 1.49)	0.676
Consumption of soft drinks^a^			0.126		
0. No	287 (92.58)	276 (89.03)		Reference	
1. Yes	23 (7.42)	34 (10.97)		1.54 (0.88, 2.68)	0.128
Ingestion of processed juice^a^			0.087		
0. No	289 (93.23)	277 (89.35)		Reference	
1. Yes	21 (6.77)	33 (10.65)		1.64 (0.93, 2.90)	0.090
Consumption of frozen food^a^			0.910		
0. No	263 (84.84)	264 (85.16)		—	—
1. Yes	47 (15.16)	46 (14.84)			
Maternal misuse of substances/drugs^b^			**0.004**		
0. No	300 (96.77)	283 (91.29)		Reference	
1. Yes	10 (3.23)	27 (8.71)		2.86 (1.36, 6.02)	**0.006**

*Note:* Bold *p* values represent the significant relation.

Abbreviations: ASD, autism spectrum disorder; cOR, crude odds ratio.

^a^ During pregnancy and postnatal 3 years.

^b^ During pregnancy.

The BLR model analysis (Table 4 and Figure 1) represents that the prenatal supplements and the sources of some dietary factors are associated with the odds of ASD and are presented as a cOR including CIs, with a *p* value. The consumption of farmed fish during pregnancy to postnatal 3 years and maternal misuse of substances or drugs during pregnancy were significantly associated with an increase in the ASD odds as compared to RG. The consumption of farmed chicken and eggs during pregnancy to postnatal 3 years and taking prenatal supplementary drugs were significantly associated with a decrease in the ASD odds as compared to RG.

### 3.3 Adjusted Effects on Prenatal Supplements and Sources of Dietary Factors

Table 5 and Figure 2 present the findings from an MLR analysis, examining the association between prenatal dietary factors, certain sociodemographic variables, and ASD in comparison with HCs. We report the results as aORs with corresponding 95% CIs and *p* values, indicating the strength and statistical significance of the associations. The model adjusts for important covariates, including paternal and maternal age groups, levels of education, SES, and the gender of the child.

**TABLE 5:** The multiple logistic regression (MLR) model analysis with the adjustment of covariates between autism and prenatal supplements and sources of some dietary factors during pregnancy to postnatal 3 years in the cases and controls, recruited from Bangladesh, 2020–2021 (*n* = 620).

Factors	Levels/Categorical	MLR model	
		aOR (95% CI)^a^	*p* value
Prenatal supplements intake	0. No 1. Yes	Reference 0.57 (0.37, 0.87)	**0.009**
Source of consumed chicken and egg^b^	0. Others 1. Firmed	Reference 0.41 (0.28, 0.61)	**<0.001**
Source of consumed vegetable^b^	0. Others 1. Farmed	Reference 0.32 (0.12, 0.85)	**0.023**
Sources of consumed fruit^b^	0. Others 1. Farmed	Reference 3.24 (1.20, 8.79)	**0.021**
Sources of consumed fish^b^	0. Others 1. Firmed	Reference 1.95 (1.33, 2.85)	**0.001**
Arsenic free drinking water^b^	0. No 1. Yes	Reference 1.03 (0.45, 2.37)	0.945
Iron free drinking water^b^	0. No 1. Yes	Reference 0.89 (0.61, 1.31)	0.561
Filtering water before drinking^b^	0. No 1. Yes	Reference 1.46 (0.97, 2.20)	0.069
Consumption of soft drinks^b^	0. No 1. Yes	Reference 1.58 (0.78, 3.22)	0.207
Ingestion of processed juice^b^	0. No 1. Yes	Reference 1.56 (0.75, 3.25)	0.235
Maternal misuse of substances/drugs^c^	0. No 1. Yes	Reference 2.67 (1.18, 6.05)	**0.018**
Father's age^d^	≤21 22–35 ≥36	Reference 0.66 (0.15, 2.90) 1.16 (0.25, 5.43)	0.580 0.851
Mother's age^d^	≤18 19–30 ≥31	Reference 0.85 (0.50, 1.46) 0.59 (0.28, 1.25)	0.561 0.169
Level of education (father)^b^	≤Primary SSC/HSC ≥Bachelor's degree	Reference 1.36 (0.76, 2.43) 0.96 (0.50, 1.85)	0.299 0.903
Level of education (mother)^b^	≤Primary SSC/HSC ≥Bachelor's degree	Reference 1.30 (0.74, 2.28) 0.96 (0.47, 1.99)	0.364 0.920
Level of SES^e^	≤10,000 BDT 10,001–20,000 BDT ≥20,001 BDT	Reference 0.45 (0.28, 0.73) 0.38 (0.22, 0.65)	**0.001** **<0.001**
Subject's gender:	Female Male	Reference 2.06 (1.42, 2.97)	**<0.001**

*Note:* Bold *p* values represent the significant relation.

Abbreviation: aOR, adjusted odds ratio.

^a^ Adjusted for paternal and maternal age groups at child's birth, their levels of education and SES during pregnancy, and subject's gender.

^b^ During pregnancy and postnatal 3 years.

^c^ During pregnancy.

^d^ At the time of child birth.

^e^ SES = Socioeconomic status, measured by monthly total household income (MTHI) during pregnancy.

One of the key findings is the significant association between prenatal supplementary drug intake and a lower risk of ASD. Mothers who took prenatal supplements had a 43% reduced odds of having a child with ASD (aOR = 0.57, 95% CI 0.37, 0.87, *p* = 0.009) compared to those who did not. For instance, consuming chicken and eggs from farmed sources was associated with a 59% reduction in ASD odds (aOR = 0.41, 95% CI 0.28, 0.61, *p *< 0.001). Likewise, consuming farmed vegetables also showed a protective effect, with a 68% lower likelihood of ASD (aOR = 0.32, 95% CI 0.12, 0.85, *p* = 0.023). However, some sources of food appeared to increase the risk of ASD. Mothers who consumed farmed fruit had more than three times the odds of ASD (aOR = 3.24, 95% CI 1.20, 8.79, *p* = 0.021) compared to those who consumed other sources of fruit. A similar pattern was observed for fish consumption, where consuming farmed fish was associated with almost double the odds of ASD (aOR = 1.95, 95% CI 1.33, 2.85, *p* = 0.001). Water‐related factors, including the use of arsenic‐ or iron‐free water and the filtration of water before drinking, did not show significant associations with ASD. For example, the use of arsenic‐ and iron‐free drinking water showed no significant effect (aOR = 1.03, 95% CI 0.45, 2.37, *p* = 0.945 and aOR = 0.89, 95% CI 0.61, 1.31, *p* = 0.561; respectively), and filtering water before drinking also did not reach statistical significance (aOR = 1.46, 95% CI 0.97, 2.20, *p* = 0.069). Similarly, the consumption of soft drinks and processed juice during pregnancy did not show statistically significant associations with ASD risk. The consumption of soft drinks and processed juice showed no significant effect (aOR = 1.58, 95% CI 0.78, 3.22, *p* = 0.207 and aOR = 1.56, 95% CI 0.75, 3.25, *p* = 0.235; respectively). An important finding in the analysis is the association between maternal misuse of substances or drugs during pregnancy and an increased risk of ASD. Mothers who misused substances had nearly three times the odds of having a child with ASD (aOR = 2.67, 95% CI 1.18, 6.05, *p* = 0.018) compared to those who did not.

### 3.4 Adjusted Effects on Sociodemographic Factors (SDFs, Table 5)

In terms of sociodemographic variables, the analysis found that parental age was not significantly associated with ASD. Both advanced father groups (aOR = 0.66, 95% CI 0.15, 2.90, *p *= 0.580 for aged 22–35 years and aOR = 1.16, 95% CI 0.25, 5.43, *p *= 0.851 for aged ≥36 years) did not show statistically significant association with ASD risk compared to fathers aged ≤21 years. Similarly, maternal age showed no significant association with ASD risk. Mothers aged 19–30 years (aOR = 0.85, 95% CI 0.50, 1.46, *p *= 0.561) and those aged ≥31 years (aOR = 0.59, 95% CI 0.28, 1.25, *p *= 0.169) did not show statistically significant differences in ASD risk compared to mothers aged ≤18 years. Father's age groups between 22 and 35 years have protective trends from developing ASD, whereas age groups of ≥36 years showing slightly higher risk for ASD occurrence. On the other hand, mother's age groups between 19 and 30 or ≥31 years have protective trends from developing ASD. Holding a bachelor's degree or above (aOR = 0.96, 95% CI 0.50, 1.85, *p *= 0.903), as well as attaining SSC/HSC education levels (aOR = 1.36, 95% CI 0.76, 2.43, *p *= 0.299) for the father, showed insignificant odds of ASD in offspring as compared to RG (≤primary). For mothers, neither SSC/HSC education (aOR = 1.30, 95% CI 0.74, 2.28, *p *= 0.364) nor bachelor's education (aOR = 0.96, 95% CI 0.47, 1.99, *p *= 0.920) showed a statistically significant association with ASD risk when compared to mothers with a primary education. Both the higher levels of parental MTHI group {10,001–20,000 BDT (aOR = 0.45, 95% CI 0.28, 0.73, *p =* 0.001) and ≥20,001 BDT (aOR = 0.38, 95% CI 0.22, 0.65, *p *< 0.001)} were significantly associated with a 55% and 62% reduced odds of ASD in offspring compared to lower income group (RG: ≤10,000 BDT), respectively. Finally, the subject's gender showed a strong and significant association with ASD. Male children were significantly more likely to be diagnosed with ASD than female children, with two‐fold odds (aOR = 2.06, 95% CI 1.42, 2.97, *p* < 0.001).

## 4 Discussion

This study presents the relationships between ASD likelihood and prenatal supplements and the sources of some dietary factors in Bangladesh. Autistic‐like features and behaviors of ASD might be increased due to the suboptimal dietary condition [22].

In this study, prenatal supplementary drug intake was associated with a decrease in the odds of ASD after adjusting the covariates in the MLR model as compared to HCs. This suggests that prenatal supplementation may have a protective effect against ASD. Self‐reported moderate (3–5 times/week) prenatal supplementation was associated with a decreased risk of ASD, and ASD has a “U”‐shaped relationship with maternal multivitamin supplementation frequency in a previous study [24]. Remarkably elevated maternal plasma folate and B_12_ levels at birth were also associated with ASD risk [24], and these findings are consistent with earlier findings. Unbalanced maternal dietary habits were associated with an increase in ASD risk, and preconception calcium supplementation for pregnancy was associated with a decrease in ASD risk [30]. In a large population‐based study, autism‐related traits were associated with gestational vitamin D deficiency, which is readily preventable with safe, cheap, and accessible supplements [31]. Another review study concluded that the effects of folic acid supplementation in pregnancy were inconsistent [32]. Schmidt et al. [33] found that mothers of ASD cases reported taking fewer iron supplements during the prenatal to breastfeeding periods. Thus, ASD risk was associated with periconceptional folic acid intake [34]. Therefore, maternal prenatal supplement intake may reduce the likelihood of ASD, which is cost‐effective.

The consumption of the farmed chicken and egg during pregnancy to 3 years of postnatal life was associated with a decreased odd of ASD in the MLR model as compared to HCs in the present study. A Saudi Arabian study suggested there are no dangers from broiler meat or eggs, as they found there were no hazardous substances (i.e., Cd, Pb, As, and Se) in broiler meat or eggs [23]. Contrarily, the liver of broiler showed the highest concentration of heavy metals, with the exception of Cr, indicating that Pb and Cd ingestion exceeded the adult recommended daily allowance (RDA) [23]. One of the Bangladeshi studies found that the majority of chicken body sections had higher levels of Cr than the recommended levels, where Mn, Fe, Cd, Cr, and Pb were greater than their tolerance limits in egg samples, and this is extremely concerning for the public's health [35]. Interestingly, another Bangladeshi study found that the levels of examined Pb and Cu in chickens were lower than the maximum allowable concentration (MAC) set by FAO/WHO and other regulatory agencies [36]. Indeed, some heavy metals (especially Pb) were associated with ASD risk based on a systematic review [12]. Due to inconsistent findings, it is recommended that manufacturers of commercial feed and farmed chicken farmers take careful consideration about using those feed items and supplements. Maintaining the recommended level of ingredients for commercial feed may help reduce the odds of ASD development.

The present study found that farmed vegetables and fruits were significantly associated with decreased and increased odds of ASD, respectively, compared to HCs. Interestingly, there is a lack of study in this field. In the project attached to the present study, it was found that proximity to agricultural land within 1 mile of residence was significantly associated with a decreased odd of ASD. Thus, farmed fruits and vegetables are safe to consume [37], and higher prenatal vegetable and fruit intake was associated with reductions in child autism‐related characteristics [38]. However, in Bangladesh, vegetables and fruits are harmful to consume as they may contain heavy metals [39], which may be due to using inorganic farming or neurotoxic preservatives [40].

In this study, the consumption of the farmed fish during pregnancy to 3 years postnatal was associated with a nearly twofold increase in the ASD odds. An analysis of some selected commercially farmed fish feeds in Bangladesh found a number of heavy metals (Pb, Cd, Cr, Cu, and Zn) in varying proportions [41]. On the other hand, another Bangladeshi study demonstrated that the concentration of heavy metals (Cd, Cr, Hg, Pb, and As) in six fish species in the Dhaleshwari River water was found below the permissible limit, and the consumption of those fish is not detrimental to humans [20]. Although the estimated daily intake (EDI) values were within the normal limit, routinely exposed fish from the Dhaleshwari River area may have dangerous health effects [42]. A population‐based study found that there was no adverse effect of prenatal total blood Hg on autism, whereas mothers ate fish (regardless of fish type) during pregnancy [43]. Prenatal exposure to methylmercury (MeHg) through fish exposure was not associated with ASD risk [25, 26]. A child development study in the Seychelles found no neurodevelopmental risk of prenatal MeHg exposure from ocean fish consumption [44]. Higher fish intake in the second trimester was associated with increased child autism traits, especially shellfish and large fish species, whereas salmon decreased that risk [45]. Therefore, adequate measures should be taken by commercial fish feed manufacturers to avoid the contamination of feed with heavy metals. Reducing the heavy metal exposure of firm fish to mothers during pregnancy and the early postnatal years may reduce the risk of ASD development.

Chronic intake of inorganic arsenic (iAs) through diet and/or drinking water is linked to an elevated risk of some unfavorable consequences, according to epidemiological research (Plain Language Summary, [46]). Drinking water free of iron and arsenic, filtering water before drinking, ingesting processed juice, and consuming soft drinks were all found to have varied and nonsignificant relationships in this study. All of the commercially produced fruit juices in Dhaka, Bangladesh, had microbiological characteristics that fell within the Gulf criteria [47]. Mango juices that are commercially available locally in Bangladesh have a moderately sufficient amount of microbiological and nutritional components, but they are not highly satisfactory for human consumption [48].

A history of substance or drug misuse during pregnancy was significantly associated with nearly three times the odds of ASD (in both LRs) compared to HCs in this study, which is similar to [4] findings. Moreover, [21] stated that a higher risk of ASD is associated with exposure to some drugs during pregnancy, which was opposed by [49].

Additionally, the male gender exhibited a significant increase of two‐fold odds in ASD risk compared to HCs, which is consistent and similar in line with findings from many previous studies [12, 13, 14, 50], while contradicting this association in other studies [51, 52]. Parental higher SES significantly decreased the likelihood of ASD in offspring in this study, suggesting that lower SES may elevate the risk and are in a similar line with one of the Swedish studies [53]. In contrast, higher SES amplified the risk of ASD found in several previous studies [50, 54, 55]. The overall income status of Bangladeshis categorized the country as low‐income before 2015 and then transitioned to a lower middle income country on July 1, 2015 (https://eias.org/policy‐briefs/towards‐the‐middle‐income‐status‐in‐bangladesh/). Thus, the variation in the prevalence of ASD risk would be based on socioeconomic/geographical differences, which might have evolved due to alterations in income groups [50, 53, 54, 55] and currency depreciation against international currencies over the last two decades in Bangladesh (https://www.icab.org.bd/publication/news/4/1001/Exchange‐Rate‐Rollercoaster‐Unraveling‐the‐Impact‐of‐Currency‐Fluctuations‐on‐Private‐Sector‐Entities:~:text=These%20factors%20have%20contributed%20to,per%20USD%20in%20January%202023.). Finally, this study indicates that higher income levels may be associated with a lower risk of ASD, potentially due to better access to healthcare services and diagnosis. However, the association may reflect complex socio‐environmental dynamics that need further exploration.

The findings of this study suggest that although some farmed foods may be protective, others, like fruit and fish, could potentially increase the risk of ASD, which warrants further investigation into potential contaminants or nutritional deficiencies in these foods. Prenatal nutrition's link to offspring's ASD risk, although not yet fully understood, holds significant promise for intervention [56]. Adequate periconceptional dietary habits are critical for proper neurodevelopment [7], and deficiencies in specific micronutrients are prevalent among pregnant women in low‐ and middle‐income countries like Bangladesh [57]. This is particularly concerning in Bangladesh, where high rates of maternal malnutrition (49.6% anemia in pregnant and 47.8% in breastfeeding women) [57] and severe pollution ([58], Preprint) may further compromise food quality and safety. A previous study even suggests widespread food contamination in Bangladesh [40], raising concerns about exposure to harmful substances. For the past few decades, Bangladesh's public health has been gravely challenged by the consumption of contaminated food [40]. The findings of the present study revealed that the higher SES group might be able to intake prenatal supplements and consume vegetables, chicken or eggs, fruits, and fish. However, toxic preservatives in fruits and fish may be alarming for public health. Given the public health concern and economic burden of autism, maternal folic acid intake may prevent the development of ASD in the offspring [59, 60]. Safety measures would be useful to gradually reduce the level of toxic heavy metals in the commercial feed ingredients of chicken, fish, fruit, vegetables, and drinking water. Therefore, strategic interventions and careful consideration of taking prenatal supplements and dietary sources during pregnancy to postnatal 3 years may reduce the development of ASD odds. These insights should strongly inform policymakers, stakeholders, governmental bodies, and private agencies in crafting effective interventions and policies aimed at reducing ASD risk in the country.

### 4.1 Strengths and Limitations

This study boasts several strengths. As the first case‐control investigation of its kind in Bangladesh, on the basis of published literature and encompassing a relatively large sample across all divisions, it contributes valuable data to the field. Notably, our findings align with prior research, strengthening the evidence that prenatal supplements potentially reduce ASD risk compared to HCs, whereas maternal substance or drug misuse during pregnancy increases ASD likelihood.

However, limitations require acknowledgement. Recruiting perfectly matched controls proved challenging due to feasibility constraints, a limitation mitigated by adjusting for potential covariates in the MLR analysis. Additionally, the absence of an Autism Diagnostic Observation Schedule (ADOS) score or a similar severity measure among studied parameters limits our understanding of symptom diversity. This stems from the recruitment method, as cases were drawn from individuals registered with ASD at the PSOSK centers. To address potential recall bias, a random sampling method was employed, and question wording was carefully considered to avoid influencing participants’ responses. Although data collection challenges in the Mymensingh and Khulna divisions resulted in some deviation from the anticipated proportion for the case group, the control collection deviated to a lesser extent due to respondent unavailability in certain divisions (Table 2). The SES, in covariation with other risk factors, could be a residual confounding that we could not analyze. Finally, the categories, frequency, and periods (preconception, prenatal, and postnatal, including the first, second, and third trimesters) of supplement intake, frequency of consumption of observed foods, and types of substance or drug abuse were not assessed. Notably, further research is needed to delve deeper into specific categories, frequencies, and periods of supplement intake, consumption patterns of observed dietary items, SES covariation, and the types of substances or drugs involved in cases of maternal misuse.

## 5 Conclusion

This study offers valuable insights into potential dietary and nutritional influences on ASD risk in the Bangladeshi context. Our findings indicate that consuming farmed chicken or eggs and vegetables during pregnancy and the first 3 years of life, along with taking prenatal supplements, is associated with a decreased likelihood of ASD compared to HCs. Conversely, increased ASD odds were observed in individuals whose mothers consumed farmed fish and fruits during the same period and who engaged in substance or drug misuse during pregnancy. The offspring's gender and higher parental SES during pregnancy were also associated with an increase and decrease in the ASD likelihood, respectively. Considering the significant economic burden and public health implications of autism, these findings suggest that promoting safe and balanced maternal dietary habits during pregnancy and the early postnatal period, alongside targeted interventions supporting adequate prenatal supplement intake, may hold promise for reducing ASD risk. Future research employing larger population‐based cohorts or nested case–control designs is necessary to further elucidate and generalize the associations observed between ASD likelihood and specific sources of some dietary factors, including prenatal supplements.

## Author Contributions

**Md. Shahid Khan**: conceptualization, methodology, resources, project administration, data collection; curation and analysis, software's, drawing graphs, writing and editing original draft, visualization. **Mohammad Alamgir Kabir**: conceptualization, methodology, reviewing and editing data analysis, supervision, validation. **Shafi Mohammad Tareq**: conceptualization, methodology, reviewing and editing draft, supervision, validation. All the authors approved the final version for publication and ensured that all aspects of the manuscript have been reviewed and discussed to be exposed with the utmost precision.

## Funding

The authors have nothing to report.

## Conflicts of Interest

The authors declare no conflicts of interest.

## Supporting information




**Supporting File 1:** puh270253‐supinfo‐0001

## Data Availability

The data that support the findings of this study are available on request from the corresponding author. The data are not publicly available for reasons of privacy or ethical restrictions.
