# Supplementary material for: Maternal Prenatal Supplements Intake and Dietary Sources and Their Relation to Autism Spectrum Disorder in the Population of Bangladesh
Source: Public Health Chall. 2026 Apr 27;5(2):e70253. doi: 10.1002/puh2.70253 (PMC13114774; doi:10.1002/puh2.70253)
Supplement: Supplementary file 1 — Supporting File 1: puh270253‐supinfo‐0001 [file PUH2-5-e70253-s001.docx]

16^th^ April, 2026

**Response to the editorial teams**

Unique manuscript ID: Public Health Challenges: 4579324.

Title: Maternal prenatal supplements intake and dietary sources and their relation to autism spectrum disorder in the population of Bangladesh

We thank the editorial teams and reviewers for accepting the manuscript after their carefully reviewing it. We are sorry that the file was in incorrect section. The submitted “Supplementary data=Questionnaire” file is not mandatory to publish. This file is not cited in text; the file was just for reviewer understanding. However, authors uploaded the file is under “Additional File for Review but Not for Publication” section in system.

Please let us know if you still have any questions or concerns about the manuscript. We will be happy to address them.

Sincerely yours,

Md. Shahid Khan,

**Corresponding author (**Manuscript ID: 4579324)
